# Impact of short-term exposure to high ambient temperature on pulmonary tuberculosis: a 5-year time-series analysis in Beijing

**DOI:** 10.3389/fpubh.2025.1672848

**Published:** 2025-12-10

**Authors:** Shirong Li, Feng Guo, Chao Wang, Rongmei Liu, Wenjie Qi

**Affiliations:** 1 Department of Infectious Disease, Beijing Friendship Hospital, Capital Medical University, Beijing, China; 2 The Second Clinical Medical College, Capital Medical University, Beijing, China; 3 Department of Research Ward, Beijing Chest Hospital, Capital Medical University, Beijing, China

**Keywords:** pulmonary tuberculosis, ambient temperature, time-series study, multi-center study, environmental epidemiology

## Abstract

**Background:**

Seasonal variation has been observed in the occurrence of pulmonary tuberculosis (PTB). However, whether this variation can be attributed to suboptimal ambient temperatures remains unclear.

**Methods:**

In this study, 30,898 PTB events were identified in Beijing, China, from 2019 to 2023. A distributed-lag non-linear model (DLNM) was utilized to assess the association of daily ambient mean temperature with PTB risk and population-attributable risks, adjusting for potential time-varying confounders.

**Results:**

The reference was the minimum morbidity temperature (MMT) of 1.1 °C. The risk of onset of PTB associated with extremely (27.7 °C), sub-extremely (25.2 °C) and moderately (22.0 °C) high temperature occurred on the concurrent day, attenuated on lag 1 day and thereafter became insignificant. The relative risks of PTB at extremely (27.7 °C), sub-extremely (25.2 °C) and moderately (22.0 °C) high temperature cumulated over lag 0–7 days were 1.92 [95% confidence interval (CI): 1.17, 3.13], 1.79 (95% CI: 1.16, 2.76), and 1.66 (95% CI: 1.12, 2.47), respectively, compared to the referent temperature (1.1 °C). Stronger associations were observed for patients who were aged ≥60 years and female. The attributable fraction (AF) of PTB due to temperatures exceeding the MMT (1.1 °C) and physiologically optimal temperature (22 °C) were 11.60 and 10.73%, respectively.

**Conclusion:**

Our study provides evidence that short-term exposure to high ambient temperature is associated with an increased risk of onset of PTB, with effects being more pronounced in females and the older adults. These findings suggest that rising temperatures could pose a substantial public health challenge for PTB control. Integrating temperature-based early warnings into public health strategies may help mitigate the impact of heat on PTB transmission.

## 1 Introduction

According to WHO statistics, 10.6 million people worldwide survived tuberculosis in 2022, reversing the decline observed until 2020. As one of the 30 high-burden countries, China accounted for 7.1% of global tuberculosis cases (1). Pulmonary tuberculosis (PTB) comprises approximately 80–90% of all tuberculosis cases and poses significant socioeconomic challenges worldwide (2). Due to its highly contagious and serious complications, PTB has garnered considerable attention in clinical settings (3). The timely detection and treatment of PTB remain paramount for tuberculosis control to reduce transmission.

Beijing, a megacity characterized by high population density and exceptional mobility, faces unique challenges in PTB control. The emergence and spread of drug-resistant tuberculosis, particularly multidrug-resistant tuberculosis, impose heightened demands on treatment and prevention efforts, resulting in substantial health and economic burdens (4, 5). Furthermore, the migrant population constitutes a key demographic for PTB incidence (6). Their inherent mobility complicates treatment adherence monitoring, standardized therapy administration, and transmission prevention.

Critically, the seasonal peak in PTB diagnoses likely reflects a mixture of new infections and, perhaps more substantially, the activation of latent tuberculosis infection (LTBI). While the incubation period for new infections can be prolonged, the transition from latent infection to active disease in already-infected individuals can be rapidly triggered by acute external stressors. This study therefore focuses specifically on quantifying the impact of short-term thermal stress as a potential trigger for acute PTB onset in the latent-infected population.

The seasonal trend in the incidence of PTB was first noted in the 1930s (7). Studies indicate that most cases of PTB are initially diagnosed during the spring and summer seasons (8, 9). This suggests that higher ambient temperatures may constitute a potential risk factor for PTB. Short-term exposure to extreme heat, even lasting only a few days, can acutely and significantly impact respiratory diseases incidence and exacerbation (10, 11). Such abrupt thermal stress not only directly compromises the respiratory immune barriers (12, 13) but may also reactivate latent *Mycobacterium tuberculosis* (Mtb) (14), triggering rapid disease progression in latently infected individuals and potentially causing new infections. Neglecting this “short-term acute” association leads to a severe underestimation of the immediate threat climate stress poses to PTB epidemics. Therefore, public health strategies must urgently integrate short-term heat events into PTB early-warning systems and enhance emergency interventions to block heat-triggered acute PTB outbreaks.

Current research lacks evidence regarding the impact of short-term thermal stress on PTB susceptibility. In this study, we conducted a time-series analysis using data from PTB patients at two tertiary hospitals in Beijing, China. Our objective aimed to quantify the association between short-term exposure to ambient temperature and the risk of onset of PTB.

## 2 Methods

### 2.1 Study area

Beijing, China’s capital, sits in North China Plain (39°56′N, 116°20′E; Supplementary Figure 1). It has four distinct seasons: hot and rainy summers, cold and dry winters, and brief springs and autumns (15). The annual average temperatures range between 10 °C and 12 °C (16).

### 2.2 Study population

The study population included primary PTB patients diagnosed from 2019 to 2023, as reported by the China Information System for Disease Control and Prevention from two tertiary hospitals in Beijing: Beijing Friendship Hospital and Beijing Chest Hospital (a designated pulmonary tuberculosis treatment hospital), both affiliated with Capital Medical University. Although the exact proportion of total PTB cases in Beijing represented by these two hospitals could not be determined due to data access restrictions, Beijing Chest Hospital—as the designated clinical center for PTB control and research—and Beijing Friendship Hospital—a major tertiary general hospital—collectively manage a substantial and representative share of PTB cases in the city. All patients diagnosed with tuberculosis were included, while those with recurrence, reinfection, or duplicate reports were excluded (as shown in Supplementary Figure 2). The patient data encompassed age, gender, initial onset date, and diagnosis date. Tuberculosis was diagnosed based on symptoms, signs, molecular findings, etiology, and imaging examinations, following the Standard of the Health Industry of the People’s Republic of China—Diagnosis for Pulmonary Tuberculosis (WS 288–2017).^1^ The date the patient reported tuberculosis-related symptoms, such as cough, fatigue, and fever, was considered the time of PTB onset.

### 2.3 Exposure assessment

The daily ambient mean temperature and dewpoint temperature during the study period were sourced from the ERA5-Land climate reanalysis product provided by the European Center for Medium-Range Weather Forecasts (ECMWF) (17), with a resolution of 0.1°. In addition, the PM_2.5_ data was obtained from the ChinaHighAirPollutants (CHAP) (18), with a spatial resolution of 0.1°. The relative humidity (RH) is calculated by the ambient mean temperature and dewpoint temperature (19), as detailed in Supplementary Method S1. These meteorological factors and air pollutant estimates were averaged to provide city-level daily exposures. To reduce variability in statistical estimates caused by limited data points at extreme ends of the exposure spectrum, the ambient temperature was truncated to the range between the 2.5th and 97.5th percentiles (−7.6 °C to 27.7 °C) for all subsequent exposure-response analyses, including the DLNM and the creation of exposure-response figures.

### 2.4 Statistical analyses

Daily ambient temperature and PM_2.5_ levels were summarized using statistical measures (Supplementary Figure 3).

The association between ambient temperature and PTB risk was analyzed using a distributed-lag non-linear model (DLNM) with negative binomial distributions. The DLNM provides a flexible framework to evaluate the varying effects across different exposure levels and lag times, effectively capturing both single-lag and cumulative-lag effects (20, 21). The cross-basis framework for this study was constructed using natural cubic splines, with 4 degrees of freedom (df) for the exposure-response dimension and 3 df for the lag-response dimension. Based on previous studies on the impact of high temperatures on respiratory diseases, we set the maximum lag days to 7 to capture the short-term lag effects of the exposure-response relationship (10, 11, 22–24). This lag specification is biologically plausible for our study focus. While primary Mtb infection has a prolonged incubation period, our study targets the acute transition from latent infection to active PTB. Such reactivation events can be rapidly triggered within days by external stressors, including thermal stress, which can impair respiratory immune barriers, provoke inflammatory responses, and potentially reactivate latent Mtb (12–14). Specifically, acute heat stress has been shown to induce DNA damage and activate the cGAS-STING signaling pathway, leading to severe airway inflammation and tissue damage within days (25). Concurrently, heat stress can upregulate pro-inflammatory cytokines like IL-4 and impair alveolar epithelial function, compromising local immune defenses (26, 27). These acute pathophysiological processes are more analogous to the exacerbation of chronic respiratory conditions than to the protracted course of new infections. Therefore, a 7-day lag period is appropriate for capturing the immediate impact of temperature on PTB onset among latently infected individuals. Furthermore, this analysis controlled for potential confounding factors such as RH, PM_2.5_ levels, days of the week, public holiday and time to control for seasonal and long-term trends (20, 21). The model was formulated as:


$$\begin{array}{l} \text{logE}[Y_{t}]=\alpha +\beta T_{t,l}+\mathrm{ns}(\mathrm{RH},\mathrm{df}=3)+\mathrm{ns}({\mathrm{PM}}_{2.5},\mathrm{df}=3)\\ +\mathrm{ns}(\text{Time},7\;{\mathrm{df}}^{*}\;\text{years})+{\mathrm{Dow}}_{t}+\text{holiday}, \end{array}$$

where T_t,l_ represents 2-dimensional cross-basis matrices, l is the maximum number of lag days, Time accounts for seasonal and long-term trends, Dow_t_ adjusts for the day of the week on day t, holiday is a binary variable controlling for public holidays and ns() denotes the natural cubic spline. In addition, 3 *dfs* was utilized for RH and PM_2.5_, and 7 *dfs* per year was used for time.

To facilitate interpretation of the results, we selected referent temperature that correspond to the lowest PTB risk in the exposure-response curve, which is defined the minimum morbidity temperature (MMT) (28). The relative risks (RRs) and 95% confidence intervals (CIs) were calculated for PTB associated with extremely (97.5th percentile), sub-extreme (90th percentile) and moderately (75th percentile) high temperature, compared to the referent temperature.

To identify the susceptible population and assess the effect modification by PM_2.5_, subgroup analyses were performed based on age (<60 or ≥60 years) and gender. The statistical differences between stratum-specific estimates were tested using two-sample z tests (29), the formula is shown in Supplementary Method S2.

In our primary analyses, we employed a backward approach within the distributed lag non-linear model (DLNM) framework to calculate the ambient temperature-attributable fraction (AF) and number (AN) of pulmonary tuberculosis (PTB) onset over the specified lag period (30). This widely used approach accounts for the temporal dimension in exposure-response associations, yielding more accurate AF estimates for complex temporal patterns (31, 32). The *AF_x,t_* and *AN_x,t_* at time *t* were calculated as follows:


$$AF_{x,t}=1-e^{\left(-\sum_{I=l_{0}}^{L}\beta_{x_{t-l},l}\right)}$$

$$AN_{x,t}=AF_{x,t}\times N_{t}$$

*N_t_* represents the total case count at time *t*. *AN_x,t_* and *AF_x,t_* denote the temperature-attributable case number and fraction at *t*, calculated from exposures to temperature level *x* at lags $l_{0}$ to *L* relative to the minimum morbidity temperature (MMT) or the physiologically optimal temperature (22 °C). Empirical 95% confidence intervals (eCIs) were derived via Monte Carlo simulation, assuming multivariate normality of the regression coefficients based on their point estimates and variance–covariance matrix (1). From 5,000 simulated samples, eCIs correspond to the 2.5th and 97.5th percentiles of the resulting distributions (30, 31).

### 2.5 Heat wave analysis

To address the potential impact of heat waves (multiple consecutive days of high temperature), we defined heat waves as periods when the daily mean temperature exceeded the 90th, 92.5th, 95th, and 97.5th percentiles for 1, 2, 3, or 4 consecutive days (33). We used generalized additive models (GAMs) with a negative binomial distribution to assess the association between heat waves and PTB risk, adjusting for relative humidity, PM_2.5_, day of the week, public holidays, and long-term trends (33).

### 2.6 Sensitivity analyses

Sensitivity analyses included: (1) Model assessment with alternative parameters: overdispersion testing, cross-basis degrees of freedom (df) optimized via Akaike information criterion, time df (4, 6, 8, 10 and 12), and both unadjusted and confounder-adjusted specifications; (2) Quantification of heat effects (extreme, sub-extreme, moderate) under varying maximum lag periods (5, 14, 21, 28, 31 days) and temperature cutoffs (70th–97.5th percentiles); (3) Interactive and stratified analyses of temperature–PM_2.5_ joint effects on PTB; (4) We restricted the study period to the year 2019 only, thereby excluding the entire COVID-19 pandemic phase (34); (5) We incorporated the COVID-19 epidemic period (2020–2023) as a binary dummy variable in the main model, similar to the adjustment for day of the week and public holidays (35) (2019: 1; 2020–2023: 0).

All statistical analyses were performed using the R software package (R Foundation for Statistical Computing, Vienna, Austria; version 4.4.1). Two-sided *p*-values less than 0.05 were considered statistically significant.

## 3 Results

### 3.1 Descriptive statistics

Data were collected from 30,898 primary PTB patients. The mean age was 50.3 years, with an SD of 19.7 years, and 58.8% of the patients were male (Table 1). PTB prevalence peaked during the spring and summer (Supplementary Table 1). The average daily temperature and RH during the study period were 13 ± 11.6 °C and 52.1 ± 19.2%. The annual average daily PM_2.5_ concentration was 33.3 ± 24.0 μg m^−3^ (Supplementary Table 2).

**Table 1:** Descriptive statistics of daily meteorologic factors and air pollutants levels in Beijing, China, 2019–2023.

Environmental factor	Mean	SD	Min	2.5th	10th	25th	50th	75th	90th	97.5th	Max
Meteorological factors											
Ambient temperature, °C	13.0	11.6	−15.9	−7.6	−4.1	2.0	14.0	23.7	25.2	27.7	31.0
Relative humidity, %	52.1	19.2	10.6	20.5	27.3	36.9	50.6	68.0	79.2	87.3	95.5
Air pollutant											
PM_2.5_, μg m^−3^	37.7	29.5	3.1	5.2	9.5	17.2	29.4	47.9	64.8	96.3	202.3

SD, standard deviation; 2.5th, the 2.5th percentile; 10th, the 10th percentile; 25th, the 25th percentile; 50th, the 50th percentile; 75th, the 75th percentile; 90th, the 90th percentile; 97.5th, the 97.5th percentile; PM_2.5_, fine particulate matter, particulate matter with aerodynamic diameters ≤ 2.5 μm.

### 3.2 Associations between ambient temperatures and PTB

**Figure 1:**
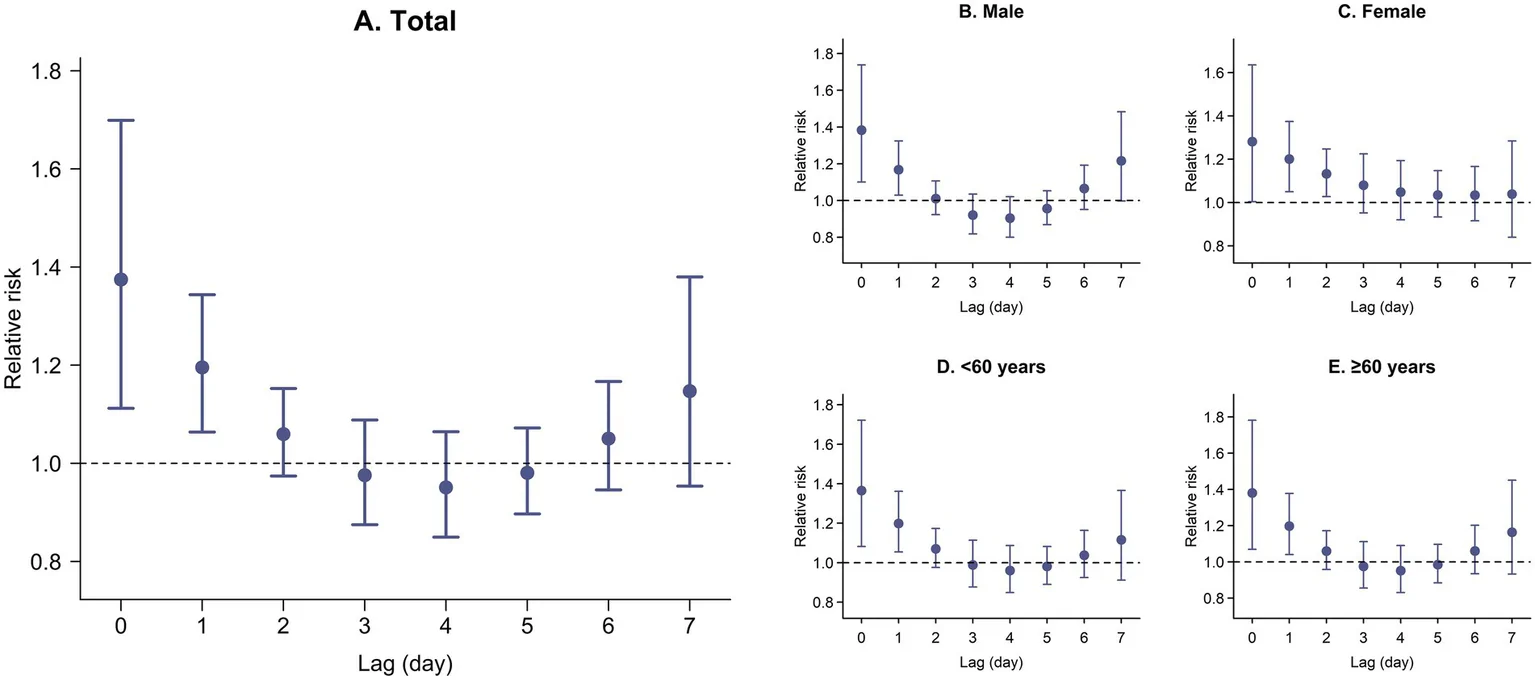
Single-lag relative risks and 95% confidence intervals of pulmonary tuberculosis at extremely high temperature^a^. ^a^Single lag-response curves are presented for: **(A)** total pulmonary tuberculosis patients; **(B)** males; **(C)** females; **(D)** patients aged <60 years; and **(E)** patients aged ≥60 years. The mean estimates of temperature-related risk attributable to extremely high temperature (27.7 °C, 97.5th percentile) are shown by purple lines, while shaded areas indicate the corresponding 95% confidence intervals.

Figure 1 showed the relative risks (RRs) of PTB associated with the effect of extremely high temperature (27.7 °C) across various lag days. The maximum risks associated with temperature was observed on the day of exposure, diminishing thereafter and becoming insignificant by lag 2 day for total PTB patients. All subgroups exhibited similar lag patterns. Notably, extremely high temperature effects persisted for 3 days among female patients. Lag–response curves for the risks of pulmonary tuberculosis at sub-extremely (25.2 °C) and moderately (22.0 °C) high temperature were shown in the Supplementary Figures 4, 5.

**Figure 2:**
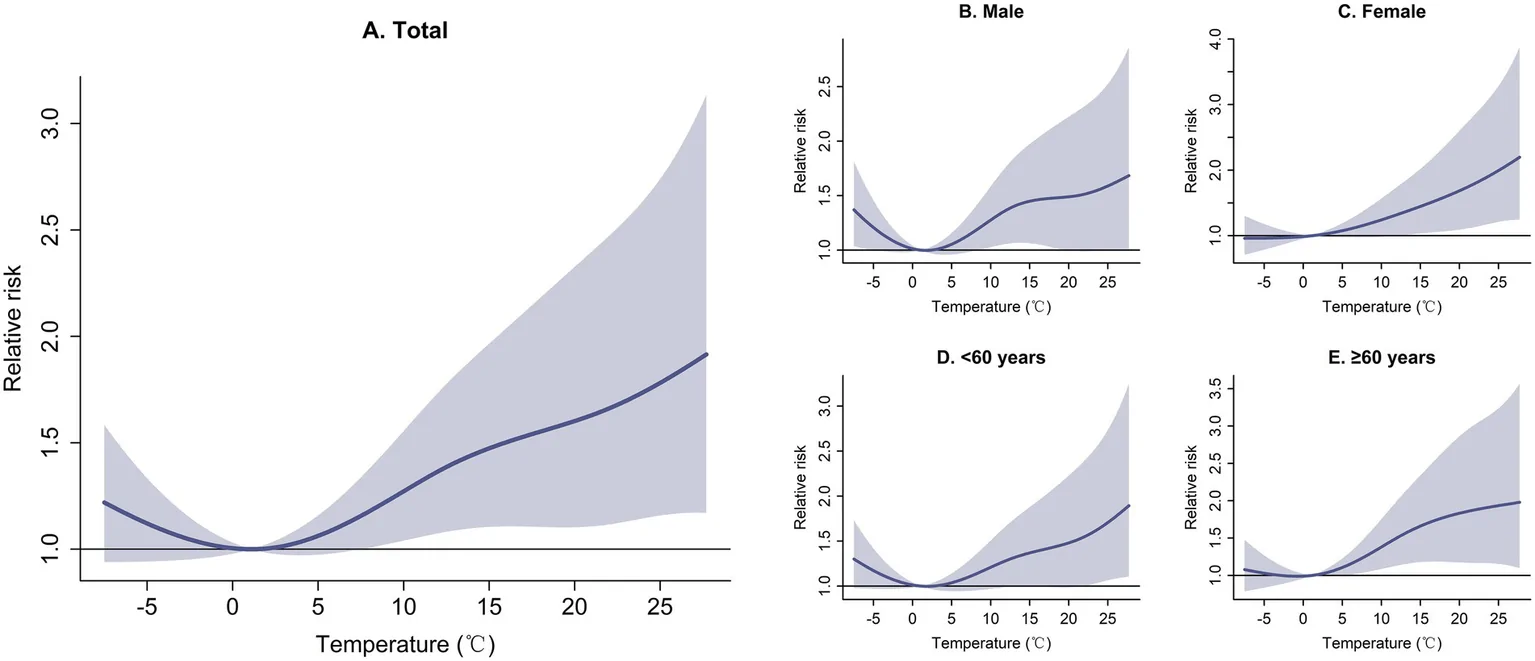
Cumulative exposure-response curves between daily mean temperature and pulmonary tuberculosis risk over 0–7 lag days^a^. ^a^Cumulative exposure-response curves are presented for: **(A)** total pulmonary tuberculosis patients; **(B)** males; **(C)** females; **(D)** patients aged <60 years; and **(E)** patients aged ≥60 years. The mean estimates of temperature-related risk are shown by purple lines, while shaded areas indicate the corresponding 95% confidence intervals. The temperature range shown on the x-axis (−7.6 °C to 27.7 °C) reflects the truncated range (2.5th to 97.5th percentiles) used in the analytical models.

V-shaped curves were observed in the exposure-response relationships between temperature and the cumulative risk of onset of PTB (Figure 2). A daily temperature of 1.1 °C corresponded to minimum PTB risk and was designated as the referent temperature. For total PTB patients, higher daily temperature was associated with increased cumulative PTB risk over lag 0–7 days, with significant RRs when temperature exceeded 7.5 °C. For instance, at extremely (29.7 °C), sub-extremely (25.2 °C) and moderately (22.0 °C) high temperature, the RR of PTB were 1.92 (95% CI: 1.17, 3.13), 1.79 (95% CI: 1.16, 2.76) and 1.66 (95% CI: 1.12, 2.47), respectively, compared to the referent temperature. Additionally, PTB risk exhibited a slight but statistically insignificant increase at temperatures below 1.1 °C. For example, at the 2.5th percentile temperature (−7.5 °C), the RR of PTB was 1.22 (95% CI: 0.94, 1.58), relative to the referent temperature. Exposure-response relationships were consistent across all subgroups.

### 3.3 Subgroup analyses

The risk of onset of PTB was further analyzed across patient subgroups categorized by sex and age. As indicated in Table 2, women exposed to extremely high (29.7 °C), sub-extremely high (25.2 °C), and moderately high (22.0 °C) temperatures exhibited significantly increased risks of PTB, with RRs of 2.20 (95% CI: 1.25, 3.87), 2.01 (95% CI: 1.22, 3.32), and 1.80 (95% CI: 1.13, 2.85), respectively. In contrast, the corresponding RRs among men were 1.68 (95% CI: 0.99, 2.86), 1.60 (95% CI: 1.00, 2.54), and 1.51 (95% CI: 0.99, 2.32), which did not reach statistical significance in the extremely and moderately high temperature groups. Similarly, patients aged over 60 years exhibited a significantly higher risk of temperature-associated PTB compared to those under 60 years. However, all differences between the subgroups did not reach statistical significance.

**Table 2:** Cumulative-lag relative risks and 95% confidence intervals over 0–7 lag days of pulmonary tuberculosis at different temperature cut-offs in sex and age groups (<60 and ≥60 years).^a^

Group	Extremeky high temperature	*p*-value	Sub-extremely high temperature	*p*-value	Moderately high temperature	*p*-value
Overall	1.92 (1.17, 3.13)		1.79 (1.16, 2.76)		1.66 (1.12, 2.47)	
Sex						
Men	1.68 (0.99, 2.86)	0.501	1.60 (1.00, 2.54)	0.510	1.51 (0.99, 2.32)	0.591
Wemen	2.20 (1.25, 3.87)		2.01 (1.22, 3.32)		1.80 (1.13, 2.85)	
Age, years						
<60	1.89 (1.10, 3.25)	0.914	1.72 (1.07, 2.76)	0.738	1.55 (1.00, 2.39)	0.557
≥60	1.98 (1.10, 3.56)		1.94 (1.15, 3.25)		1.88 (1.17, 3.02)	

df, degrees of freedom.

a Relative risks were calculated as cumulative-lag effects at three temperature thresholds relative to the minimum morbidity temperature (MMT, 1.1°C): extremely (97.5th percentile, 27.7 °C), sub-extremely (90th percentile, 25.2 °C), and moderately (75th percentile, 22.0°C) high temperature.

### 3.4 PTB risk attributable to nonoptimum temperatures

Table 3 shows the AF and AN of PTB patients due to non-optimum temperature. The AF of PTB due to temperatures exceeding the MMT (1.1 °C) and physiologically optimal temperature (22 °C) were 11.60 and 10.73%, respectively, in the overall patients. Stratified analyses revealed higher AFs among male patients (12.10 and 11.27%) compared to females (9.01 and 8.39%). Similarly, patients aged ≥60 years exhibited substantially elevated AFs (14.41 and 13.46%) relative to those <60 years (10.49 and 9.76%). Within our dataset, an estimated 2,355 and 709 cases of PTB were attributable to temperatures exceeding the MMT and physiologically optimal temperature, respectively.

**Table 3:** Attributable fractions (%, means, and 95% confidence intervals) and numbers of pulmonary tuberculosis onset attributable to non-optimum temperature.

Group	AF		AN	
	Overall^a^	Heat^b^	Overall^a^	Heat^b^
Overall	11.60 (8.91, 35.46)	10.73 (8.34, 33.17)	2,355	709
Sex				
Men	12.10 (5.77, 36.79)	11.27 (5.37, 34.57)	1,428	427
Women	9.01 (6.43, 34.17)	8.39 (6.01, 32.05)	971	237
Age, years				
<60	10.49 (4.55, 35.22)	9.76 (4.21, 33.09)	1,305	399
≥60	14.41 (10.95, 40.74)	13.46 (10.28, 38.22)	1,060	306

AF, attributable fraction; AN, attributable number.

a All non-optimal temperatures higher than the reference temperature.

b Temperatures greater than the physiologically optimal temperature threshold (22 °C).

### 3.5 Associations between heat waves and PTB

We evaluated the impact of heat waves on PTB risk using various definitions based on temperature thresholds (90th, 92.5th, 95th, and 97.5th percentiles) and durations (1–4 consecutive days). No statistically significant associations were observed between heat waves and PTB incidence across all definitions (Supplementary Figure S6). These results indicate that heat waves, as defined in this study, did not significantly contribute to PTB risk in our population.

### 3.6 Sensitivity analysis

Models were constructed based on overdispersion testing and the Akaike information criterion (Supplementary Tables S3, S4). Results demonstrated robustness across varying model parameters and onset date definitions (Supplementary Tables S5–S9). Neither multiplicative nor additive interactions between temperature and PM_2.5_ on PTB were observed (Supplementary Table S10). The robustness of our primary findings was further confirmed through sensitivity analyses addressing the potential confounding effect of the COVID-19 pandemic. Restricting the analysis to the pre-pandemic year of 2019 (Supplementary Figure S7) and adjusting for the COVID-19 period as a dummy variable (Supplementary Figure S8) yielded exposure-response relationships consistent with those of the main model. The overall shape of the temperature-PTB association and the position of the minimum morbidity temperature remained largely unchanged, indicating that the observed association between high temperature and PTB risk is not substantially confounded by the pandemic.

## 4 Discussion

### 4.1 Justification for the short-term lag period

A key consideration in our study is the rationale for selecting a 7-day maximum lag period to assess short-term temperature effects. We acknowledge that the incubation period for primary TB infection is typically prolonged and that direct evidence on the time course of LTBI reactivation following environmental stressors is limited. However, our analysis primarily reflects the acute reactivation of LTBI rather than the acquisition of new infection. Substantial evidence suggests that short-term thermal stress can act as a potent trigger for such reactivation. High temperatures have been shown to induce hyperthermia-associated DNA damage and activate the cGAS-STING signaling pathway, leading to severe airway inflammation and tissue damage within days (25). Concurrently, heat stress can upregulate pro-inflammatory cytokines like IL-4 and impair alveolar epithelial function, compromising local immune defenses (26, 27). These immunological and parenchymal disruptions can create a permissive environment for the reactivation of dormant Mtb, a process that can manifest clinically within days—a timeframe consistent with the acute exacerbation patterns observed in other respiratory conditions influenced by temperature (10, 11). Our observed risk peaks at lag 0 and 1 days, with rapid attenuation thereafter, strongly support this paradigm of an acute triggering event. Consequently, the 7-day lag window is clinically and biologically relevant for investigating how short-term heat exposure precipitates active disease in the substantial latently infected population.

### 4.2 Principal findings

The cornerstone of tuberculosis management involves the early detection and treatment of PTB to minimize transmission. Our multi-center time series analysis examined the association between daily ambient temperature and the prevalence of primary PTB. We found that short-term exposure to high ambient temperatures may amplify the risk of primary PTB. The complex relationship and lag patterns of short-term exposure to ambient temperature and PTB risk remain underexplored in existing research. Our insights could inform clinical PTB management strategies and the crafting of precise public health policies.

### 4.3 Comparison with other studies

Our study demonstrates that short-term exposure to higher daily temperatures is linked with elevated PTB risk, which may explain the spring and summer predominance of PTB incidence consistently observed in epidemiological studies (8, 9, 36). Moreover, our findings are consistent with earlier research examining the association between ambient temperature and tuberculosis (37–39). However, studies across various cities report contrary findings. Research in Shandong, Eastern China, and Urumqi uniformly linked high temperatures to reduced PTB risk, with Urumqi specifically observing this beyond a temperature threshold (40–42). This apparent contradiction may stem from the selection of lag-time frameworks. Prior studies predominantly used lag windows exceeding 3 months. Yet, long-lag analyses face significant limitations, including exposure measurement error, ecological fallacy, uncontrolled confounding, and overadjustment, which hinder translation into actionable public health interventions (43–46). Therefore, to facilitate early warning systems and rapid intervention strategies for PTB, this study investigates the impact of short-term exposure to higher temperatures on PTB incidence risk.

Furthermore, the risk of heat-related PTB can persist for up to 2 days after exposure, with the greatest risk occurring on the corresponding day. This corresponds to the established lag pattern of high temperature effects on respiratory diseases (47, 48). Thus, immediate measures are crucial to prevent PTB outbreaks following days marked by high, rising, or volatile temperatures.

The biological mechanisms underlying these associations remain uncertain, and the present study does not provide direct evidence. Several mechanisms elucidate how high temperature influences the risk of onset of PTB. Firstly, high temperatures enhance the pathogenicity of Mtb (14). Elevated ambient temperatures improve the stability of tuberculin protein. This enhancement promotes the survival and pathogenicity of Mtb within the host (49). Secondly, the post-transcriptional modifiers of Mtb exhibit strong stability at high temperatures, thereby maintaining the stability, translation efficiency, and functional properties of Mtb in the host (50). Thirdly, higher temperatures has been linked to reductions in lung function (26). Studies have demonstrated that high temperatures exacerbate airway inflammation in animal models by upregulating pro-inflammatory cytokines such as IL-4 (26, 27). Hyperthermia-induced DNA damage also activates the cGAS-STING signaling pathway, leading to severe airway or lung tissue damage (25), manifesting as alveolar collapse, vascular congestion, and neutrophil infiltration (26).

In our analysis, we observed that women are more susceptible to heat and warmer temperatures, a finding consistent with previous studies that may be related to differences in sensitivity and resilience (51). It potentially due to high-temperature stress impairing estrogen’s regulatory role in immune cell function. Additionally, women generally have smaller body size and surface area, lower body weight, height, and maximal oxygen consumption, resulting in lower heat exchange efficiency and greater difficulty adapting to heat (52, 53). While the older adults is generally more vulnerable to heat (54), likely attributable to an age-related decline in baseline immune function, reduced thermoregulatory and cardiopulmonary capacity, and a higher prevalence of underlying health conditions (55).

This study demonstrates for the first time that non-optimal temperatures significantly contribute to PTB risk, with heat (>22 ℃) being the predominant driver. Overall, 11.60% of PTB cases were attributable to non-optimal temperatures, corresponding to 2,355 excess cases. Notably, heat exposure alone accounted for 10.73% of this burden, representing 709 attributable cases. To our knowledge, no previous study has quantified the PTB burden attributable to non-optimal temperatures. Both a study in a subtropical city in China and one in Hong Kong reported similar AFs of incidence for total respiratory diseases attributable to high temperature (7.5 and 10.7%, respectively) (11, 56). The heterogeneity was mainly due to differences in outcomes (incidence vs. mortality) and estimations of exposure–response relationships. Despite statistically significant point estimates, the wide 95% confidence intervals across all attributable fractions (AFs) indicate substantial uncertainty in the magnitude of effect (57, 58). These intervals (e.g., 8.91–35.46% for overall AF) suggest that while a non-trivial temperature-attributable burden exists, the precise risk quantification warrants further investigation with larger datasets or refined exposure assessments. It is also important to consider that individuals who are vulnerable to heat for various reasons may be hospitalized more frequently and therefore have a greater likelihood of being diagnosed with tuberculosis.

### 4.4 Comparison with heat wave effects

While heat waves are often considered more harmful than single hot days in environmental health studies (46), our additional analysis did not find significant associations between heat waves and PTB risk. This may reflect the acute nature of temperature effects on PTB, as evidenced by our main findings where the risk peaked on the same day and attenuated rapidly. The lack of a cumulative effect from prolonged heat exposure could be due to the specific pathophysiology of PTB reactivation, which may be triggered by immediate thermal stress rather than sustained exposure (12). Additionally, the climate in Beijing, characterized by distinct seasons and relatively short heat waves (58), might not provide sufficient prolonged exposure to impact PTB risk (25). Further studies in regions with more extreme and prolonged heat waves are needed to clarify these relationships (43).

### 4.5 Strengths and limitations

We conducted a time-series analysis to control for time-varying covariates such as long-term trends, seasonality, day of the week, RH, and PM_2.5_. This approach also included an evaluation of the impact of air pollutants on the relationship between temperature and mortality. Our results suggest that higher temperatures may be a potential risk factor for PTB. This finding could facilitate early detection of PTB, enhance risk management, and inform preventative measures, such as using air conditioning. In global warming, these insights are particularly valuable. Furthermore, we performed sensitivity analyses to evaluate the potential impact of the COVID-19 pandemic on our results (34, 35). The consistency of the temperature-PTB association in models restricted to the pre-pandemic year 2019 and in models adjusting for the pandemic period as a covariate reinforces the robustness of our primary findings. This suggests that the observed short-term effect of high temperature on PTB risk is independent of the substantial societal and healthcare disruptions caused by the pandemic.

However, this study is subject to several limitations that must be acknowledged. Firstly, we did not adjust for individual-level confounding factors, which could influence the results. Secondly, the data were limited to two hospitals. Although these institutions collectively manage a substantial share of PTB cases in Beijing, the inclusion of a specialized tuberculosis hospital may have led to an overrepresentation of severe cases, potentially limiting the generalizability of our findings to milder or community-managed PTB cases. Thirdly, the study utilized the average temperature of Beijing instead of individual-level temperature exposures, potentially introducing measurement errors into our analysis. Fourth, this is an ecological study using city-level aggregated data. The associations observed between ambient temperature and PTB risk are at the population level and should not be directly inferred to individuals. Fifth, due to the unavailability of individual residential addresses, we assigned city-level average exposure based on Beijing meteorological data. Although we excluded patients transferred from other hospitals, we cannot entirely rule out exposure misclassification for non-residents. However, given the acute reactivation focus of our study, most patients likely sought care locally, mitigating some of this concern. Moreover, despite our use of a time-series design and adjustment for multiple confounders, the study remains observational in nature and cannot establish causal relationships.

## 5 Conclusion

Higher temperature is linked with an increased risk of onset of PTB. The risk associated with these temperature variables begins on the day of exposure and extends for 1 day. Given the non-specific early symptoms of PTB, identifying environmental risk factors is crucial for the early detection, effective treatment, and prevention of PTB.

## Data Availability

The raw data supporting the conclusions of this article will be made available by the authors without undue reservation.

## Footnotes

1
www.nhc.gov.cn/wjw/wsbzxx/wsbz.shtml
