# Correction: Impact of short-term exposure to high ambient temperature on pulmonary tuberculosis: a 5-year time-series analysis in Beijing

**DOI:** 10.3389/fpubh.2026.1855737

**Published:** 2026-08-07

**Authors:** Shirong Li, Feng Guo, Chao Wang, Rongmei Liu, Wenjie Qi

**Affiliations:** 1 Department of Infectious Disease, Beijing Friendship Hospital, Capital Medical University, Beijing, China; 2 The Second Clinical Medical College, Capital Medical University, Beijing, China; 3 Department of Research Ward, Beijing Chest Hospital, Capital Medical University, Beijing, China

**Keywords:** pulmonary tuberculosis, ambient temperature, time-series study, multi-center study, environmental epidemiology

Throughout the text, all instances of “risk of PTB” were corrected to “risk of onset of PTB”.

The Equation in the last paragraph of *2.4 Statistical analyses* was erroneously given as AF_*x, t*_ = 1 - e^(−σi=0∧L β_*x, t*_). The correct equation is *AFx,t* = 1 $-\;e^{\left(-\overset{L}{\underset{l=l_{0}}{\sum }}\beta_{x_{t-l},l}\;\right)}$.

A correction has been made to Section *2.2 Study population*. The corrected paragraph reads:

“The study population included primary PTB patients diagnosed from 2019 to 2023, as reported by the China Information System for Disease Control and Prevention from two tertiary hospitals in Beijing: Beijing Friendship Hospital and Beijing Chest Hospital (a designated pulmonary tuberculosis treatment hospital), both affiliated with Capital Medical University. Although the exact proportion of total PTB cases in Beijing represented by these two hospitals could not be determined due to data access restrictions, Beijing Chest Hospital—as the designated clinical center for PTB control and research—and Beijing Friendship Hospital—a major tertiary general hospital—collectively manage a substantial and representative share of PTB cases in the city. All patients diagnosed with tuberculosis were included, while those with recurrence, reinfection, or duplicate reports were excluded (as shown in Supplementary Figure 2). The patient data encompassed age, gender, initial onset date, and diagnosis date. Tuberculosis was diagnosed based on symptoms, signs, molecular findings, etiology, and imaging examinations, following the Standard of the Health Industry of the People's Republic of China—Diagnosis for Pulmonary Tuberculosis (WS 288–2017).^**1**^ The date the patient reported tuberculosis-related symptoms, such as cough, fatigue, and fever, was considered the time of PTB onset.”

A correction has been made to the Section *4.3 Comparison with other studies*, paragraph 3. The corrected paragraph reads:

“The biological mechanisms underlying these associations remain uncertain, and the present study does not provide direct evidence. Several mechanisms elucidate how high temperature influences the risk of onset of PTB. Firstly, high temperatures enhance the pathogenicity of Mtb (14). Elevated ambient temperatures improve the stability of tuberculin protein. This enhancement promotes the survival and pathogenicity of Mtb within the host (49). Secondly, the post-transcriptional modifiers of Mtb exhibit strong stability at high temperatures, thereby maintaining the stability, translation efficiency, and functional properties of Mtb in the host (50). Thirdly, higher temperatures has been linked to reductions in lung function (26). Studies have demonstrated that high temperatures exacerbate airway inflammation in animal models by upregulating pro-inflammatory cytokines such as IL-4 (26, 27). Hyperthermia-induced DNA damage also activates the cGAS-STING signaling pathway, leading to severe airway or lung tissue damage (25), manifesting as alveolar collapse, vascular congestion, and neutrophil infiltration (26).”

A correction has been made to the Section *4.3 Comparison with other studies*, paragraph 5. The corrected paragraph reads:

“This study demonstrates for the first time that non-optimal temperatures significantly contribute to PTB risk, with heat (>22 °C) being the predominant driver. Overall, 11.60% of PTB cases were attributable to non-optimal temperatures, corresponding to 2,355 excess cases. Notably, heat exposure alone accounted for 10.73% of this burden, representing 709 attributable cases. To our knowledge, no previous study has quantified the PTB burden attributable to non-optimal temperatures. Both a study in a subtropical city in China and one in Hong Kong reported similar AFs of incidence for total respiratory diseases attributable to high temperature (7.5 and 10.7%, respectively) (11, 56). The heterogeneity was mainly due to differences in outcomes (incidence vs. mortality) and estimations of exposure–response relationships. Despite statistically significant point estimates, the wide 95% confidence intervals across all attributable fractions (AFs) indicate substantial uncertainty in the magnitude of effect (57, 58). These intervals (e.g., 8.91–35.46% for overall AF) suggest that while a non-trivial temperature-attributable burden exists, the precise risk quantification warrants further investigation with larger datasets or refined exposure assessments. It is also important to consider that individuals who are vulnerable to heat for various reasons may be hospitalized more frequently and therefore have a greater likelihood of being diagnosed with tuberculosis.”

The original version of this article has been updated.

