# Supplementary material for: Impact of short-term exposure to high ambient temperature on pulmonary tuberculosis: a 5-year time-series analysis in Beijing
Source: Front Public Health. 2025 Dec 10;13:1672848. doi: 10.3389/fpubh.2025.1672848 (PMC12727902; doi:10.3389/fpubh.2025.1672848)
Supplement: Supplementary file 1 [file Data_Sheet_1.pdf]

Supplemental Material

High Ambient Temperature and Pulmonary Tuberculosis: A 5-Year Time-Series Analysis in Beijing

Shirong Li[1†], Feng Guo[2†], Chao Wang[1], Rongmei Liu[4*], Wenjie Qi[1*]

[†] Shirong Li and Feng Guo contributed equally to this work.

[1] Department of Infectious Disease, Beijing Friendship Hospital, Capital Medical University, Beijing, China

[2] The second clinical medical college, Capital Medical University, Beijing, China

[3] Department of Research Ward, Beijing Chest Hospital, Capital Medical University, Beijing, China

[*]Address for correspondence:

Dr. Rongmei Liu, Department of Research Ward, Beijing Chest Hospital, Capital Medical University, No. 97 Ma Chang, Tongzhou District, Beijing 101149, China. E-mail: Lrongmei@163.com;

Dr. Wenjie Qi, Department of Infectious Disease, Beijing Friendship Hospital, Capital Medical University, No.95 Yong 'an Road, Xicheng District, Beijing 100050, China. E-mail: qi_wenjie@ccmu.edu.cn.

Supplemental Methods

Supplementary Methods S1. Calculation of relative humidity.

Supplementary Methods S2. Assessment of effect modification for stratification analysis.

Supplemental Tables

Supplemental Table S1. Descriptive statistics of the study population and pulmonary tuberculosis patients.

Supplemental Table S2. pulmonary tuberculosis cases stratified by season, age, and sex.

Supplemental Table S3. Model diagnostic tests for overdispersion in the association between PTB onset and high temperature exposures.

Supplemental Table S4. Akaike information criterion values for selected degrees of freedom in temperature and lag spaces of distributed lag nonlinear models.

Supplemental Table S5. Cumulative-lag relative risks and 95% confidence intervals over 0 to 7 lag days of pulmonary tuberculosis at different temperature cut-offs by changing the degrees of freedom for time (4, 6, 8, 10 and 12) per year.

Supplemental Table S6. Cumulative-lag relative risks and 95% confidence intervals over 0 to 7 lag days of pulmonary tuberculosis at different temperature cut-offs in the unadjusted and confounder-adjusted models.

Supplemental Table S7. Single-lag relative risks and 95% confidence intervals of pulmonary tuberculosis at different temperature cut-offs by changing the maximum lag period (14, 21, 28, and 31 days).

Supplemental Table S8. Cumulative-lag relative risks and 95% confidence intervals of pulmonary tuberculosis at different temperature cut-offs by changing the maximum lag period (14, 21, 28, and 31 days).

Supplemental Table S9. Cumulative-lag relative risks and 95% confidence intervals over 0 to 7 lag days for pulmonary tuberculosis associated with high temperatures using different temperature cutoffs (from the 97.5^{th} to the 70th percentile).

Supplemental Table S10. Interactive and stratified analyses of temperature and $PM_{2.5}$ on pulmonary tuberculosis.

Supplemental Figures

Supplemental Figure S1. Geographical location and climate characteristics of Beijing.

Supplemental Figure S2. Flow diagram for the inclusion and exclusion of pulmonary tuberculosis patients.

Supplemental Figure S3. Time-series plots of daily pulmonary tuberculosis events, meteorological factors, and $PM_{2.5}$ levels in Beijing, China, 2019−2023.

Supplemental Figure S4. Single-lag relative risks and 95% confidence intervals of pulmonary tuberculosis at sub-extremely high temperature.

Supplemental Figure S5. Single-lag relative risks and 95% confidence intervals of pulmonary tuberculosis at moderately high temperature.

Supplemental Figure S6. Association of exposure to heat waves with pulmonary tuberculosis.

Supplemental Figure S7. Cumulative exposure-response curves between daily mean temperature and pulmonary tuberculosis risk over 0 to 7 lag days in 2019.

Supplemental Figure S8. Cumulative exposure-response curves between daily mean temperature and pulmonary tuberculosis risk over 0 to 7 lag days after adjusting the impact of the COVID-19.

Supplemental Methods

Supplementary Methods S1. Calculation method of relative humidity.

The relative humidity (RH) is calculated by the ambient mean temperature (T) and dewpoint temperature (DT) using the following equation [1]:

RH = 100*(exp((17.625*DT)/ (243.04 + DT)) / exp ((17.625*T)/(243.04 + T)))

This method exhibits a relative error not exceeding 0.384% within the temperature range of −40°C to 50°C [1].

Supplementary Methods S2. Assessment of effect modification for stratification analysis.

The statistical differences between stratum-specific estimates were tested using two-sample z tests with the following formula:

$(\hat{Q}_1 - \hat{Q}_2) / \sqrt{(\widehat{SE}_1^2 + \widehat{SE}_2^2)}$

where $\hat{Q}_1$ and $\hat{Q}_2$ are regression coefficients by strata, and $\widehat{SE}_1^2$ and $\widehat{SE}_2^2$ represent their standard errors [2].

Supplemental Tables

Supplemental Table S1. Descriptive statistics of the study population and pulmonary tuberculosis patients.

Variables	Value
Total PTB counts, n (%)	30,898 (100.0)
Age, mean (SD), year	50.3 (19.7)
Age group, n (%)	
<60 years	19,214 (62.2)
≥60 years	10,694 (34.6)
Sex, n (%)	
Men	18,155 (58.8)
Women	12,743 (41.2)

SD, standard deviation.

Supplemental Table S2. pulmonary tuberculosis cases stratified by season, age, and sex.

Characteristics	Spring	Summer	Autumn	Winter
Overall	7969	8322	7632	6975
Age				
<60	4759	4792	4469	4135
≥60	3210	3530	3163	2840
Sex				
Male	4922	5157	4727	4408
Female	2757	2868	2716	2353

Supplemental Table S3. Model diagnostic tests for overdispersion in the association between PTB onset and high temperature exposures.

Model	Deviance	df.residual	Deviance / df.residual	*P* value [a]
Poisson regression	11065	1763	6.28	<0.001
Quasi-Poisson regression	11065	1763	6.28	<0.001
Negative Binomial regression	1738	1540	1.13	<0.001

df, degrees of freedom.

[a] P<0.05 indicates overdispersion.

Supplemental Table S4. Akaike information criterion values for selected degrees of freedom in temperature and lag spaces of distributed lag nonlinear models.

df for temperature	df for lag	AIC
3	3	10546.3
3	4	10549.7
3	5	10552.0
3	6	10554.6
4	3	10545.3*
4	4	10550.5
4	5	10552.0
4	6	10555.2
5	3	10548.1
5	4	10555.4
5	5	10557.3
5	6	10563.8
6	3	10550.4
6	4	10559.7
6	5	10561.2
6	6	10567.7

df, degrees of freedom; AIC, Akaike information criterion.

The asterisk denotes the model configuration with optimal fit, as determined by minimal AIC value.

Supplemental Table S5. Cumulative-lag relative risks and 95% confidence intervals over 0 to 7 lag days of pulmonary tuberculosis at different temperature cut-offs by changing the degrees of freedom for time (4, 6, 8, 10 and 12) per year.[a]

df for time	Extremely high temperature	Sub-extremely high temperature	Moderately high temperature
df=4	1.75 (1.10,2.81)	1.70 (1.13,2.58)	1.65 (1.15,2.36)
df=6	2.08 (1.28,3.40)	1.89 (1.25,2.88)	1.70 (1.19,2.43)
df=8	1.83 (1.11,3.01)	1.76 (1.13,2.74)	1.67 (1.11,2.52)
df=10	1.63 (1.01,2.70)	1.62 (1.04,2.52)	1.57 (1.04,2.36)
df=12	1.77 (1.03,3.05)	1.75 (1.08,2.83)	1.69 (1.07,2.66)

df, degrees of freedom.

[a] Relative risks were calculated as cumulative-lag effects at three temperature thresholds relative to the minimum morbidity temperature (MMT, 1.1°C): extremely (97.5th percentile, 27.7°C), sub-extreme (90th percentile, 25.2°C), and moderately (75th percentile, 22.0°C) high temperature.

Supplemental Table S6. Cumulative-lag relative risks and 95% confidence intervals over 0 to 7 lag days of pulmonary tuberculosis at different temperature cut-offs in the unadjusted and confounder-adjusted models.[a]

	Extremely high temperature	Sub-extremely high temperature	Moderately high temperature
Model 1 [b]	1.88 (1.16,3.05)	1.76 (1.15,2.69)	1.63 (1.10,2.40)
Model 2 [b]	1.92 (1.17,3.13)	1.79 (1.16,2.76)	1.66 (1.12,2.47)
Model 3 [b]	1.83 (1.08,3.08)	1.73 (1.08,2.75)	1.62 (1.05,2.49)

[a] Relative risks were calculated as cumulative-lag effects at three temperature thresholds relative to the minimum morbidity temperature (MMT, 1.1°C): extremely (97.5th percentile, 27.7°C), sub-extreme (90th percentile, 25.2°C), and moderately (75th percentile, 22.0°C) high temperature.

[b] model 1: adjusted for time and day of the week; model 2: model 1 + adjusted for relative humidity and $PM_{2.5}$; model 3: model 2 + adjusted for temperature × $PM_{2.5}$.

Supplemental Table S7. Single-lag relative risks and 95% confidence intervals of pulmonary tuberculosis at different temperature cut-offs by changing the maximum lag period (14, 21, 28, and 31 days).[a]

The maximum lag period		Lag 0 day	Lag 1 day	Lag 2 day	Lag 3 day	Lag 4 day	Lag 5 day	Lag 6 day	Lag 7 day
Extremely high temperature	14 days	1.21 (1.05,1.39)	1.16 (1.04,1.29)	1.11 (1.02,1.20)	1.07 (1.00,1.14)	1.04 (0.98,1.10)	1.01 (0.95,1.07)	0.99 (0.92,1.06)	0.98 (0.91,1.06)
	21 days	1.11 (1.00,1.24)	1.10 (1.01,1.20)	1.09 (1.01,1.17)	1.08 (1.01,1.15)	1.07 (1.01,1.13)	1.05 (1.00,1.11)	1.05 (0.99,1.10)	1.04 (0.99,1.09)
	28 days	1.10 (1.01,1.20)	1.10 (1.01,1.19)	1.09 (1.01,1.17)	1.09 (1.02,1.16)	1.08 (1.02,1.15)	1.08 (1.02,1.14)	1.08 (1.02,1.13)	1.07 (1.02,1.13)
	31 days	1.09 (1.01,1.19)	1.09 (1.01,1.18)	1.09 (1.01,1.17)	1.09 (1.02,1.16)	1.08 (1.02,1.15)	1.08 (1.02,1.14)	1.08 (1.02,1.14)	1.07 (1.02,1.13)
Sub-extremely high temperature	14 days	1.15 (1.01,1.30)	1.11 (1.01,1.22)	1.08 (1.01,1.16)	1.06 (1.00,1.12)	1.03 (0.98,1.09)	1.01 (0.96,1.07)	1.00 (0.94,1.07)	0.99 (0.93,1.06)
	21 days	1.10 (1.00,1.21)	1.09 (1.00,1.18)	1.08 (1.01,1.15)	1.07 (1.01,1.13)	1.05 (1.00,1.11)	1.05 (1.00,1.09)	1.04 (0.99,1.08)	1.03 (0.98,1.08)
	28 days	1.09 (1.00,1.18)	1.08 (1.01,1.16)	1.08 (1.01,1.15)	1.08 (1.01,1.14)	1.07 (1.02,1.13)	1.07 (1.02,1.12)	1.07 (1.02,1.12)	1.06 (1.02,1.11)
	31 days	1.07 (1.00,1.16)	1.07 (1.00,1.15)	1.07 (1.01,1.14)	1.07 (1.01,1.14)	1.07 (1.02,1.13)	1.07 (1.02,1.12)	1.07 (1.02,1.12)	1.07 (1.02,1.12)
Moderately high temperature	14 days	1.09 (1.00,1.22)	1.08 (1.00,1.17)	1.06 (0.99,1.13)	1.04 (0.99,1.10)	1.03 (0.98,1.08)	1.02 (0.97,1.07)	1.01 (0.95,1.07)	1.01 (0.95,1.07)
	21 days	1.09 (1.00,1.18)	1.07 (1.00,1.16)	1.06 (1.00,1.13)	1.05 (1.00,1.11)	1.04 (1.00,1.09)	1.03 (0.99,1.08)	1.03 (0.99,1.07)	1.02 (0.98,1.06)
	28 days	1.07 (1.00,1.15)	1.07 (1.00,1.14)	1.07 (1.00,1.13)	1.06 (1.01,1.12)	1.06 (1.01,1.11)	1.06 (1.01,1.11)	1.06 (1.02,1.10)	1.06 (1.02,1.10)
	31 days	1.06 (1.00,1.13)	1.06 (1.00,1.13)	1.06 (1.00,1.12)	1.06 (1.01,1.12)	1.06 (1.01,1.11)	1.06 (1.02,1.11)	1.06 (1.02,1.11)	1.06 (1.02,1.10)

[a] Relative risks were calculated as cumulative-lag effects at three temperature thresholds relative to the minimum morbidity temperature (MMT, 1.1°C): extremely (97.5th percentile, 27.7°C), sub-extreme (90th percentile, 25.2°C), and moderately (75th percentile, 22.0°C) high temperature.

Supplemental Table S8. Cumulative-lag relative risks and 95% confidence intervals of pulmonary tuberculosis at different temperature cut-offs by changing the maximum lag period (14, 21, 28, and 31 days).

The maximum lag period	Extremely high temperature	Sub-extremely high temperature	Moderately high temperature
14 days	2.30 (1.12,4.75)	2.05 (1.09,3.83)	1.81 (1.03,3.16)
21 days	2.77 (1.05,7.27)	2.67 (1.17,6.11)	2.53 (1.23,5.19)
28 days	5.97 (1.69,21.14)	5.59 (1.86,16.78)	5.08 (1.94,13.28)
31 days	6.79 (1.70,27.17)	6.26 (1.86,21.10)	5.67 (1.95,16.49)

[a] Relative risks were calculated as cumulative-lag effects at three temperature thresholds relative to the minimum morbidity temperature (MMT, 1.1°C): extremely (97.5th percentile, 27.7°C), sub-extreme (90th percentile, 25.2°C), and moderately (75th percentile, 22.0°C) high temperature.

Supplemental Table S9. Cumulative-lag relative risks and 95% confidence intervals over 0 to 7 lag days for pulmonary tuberculosis associated with high temperatures using different temperature cutoffs (from the 97.5th to the 70th percentile).

Cutoffs [a]	Temperature (°C)	RR (95% CI)
97.5th percentiles	27.7	1.92 (1.17,3.13)
95th percentiles	26.6	1.86 (1.17,2.95)
92.5th percentiles	26.8	1.82 (1.17,2.84)
90th percentiles	25.2	1.79 (1.16,2.76)
87.5th percentiles	24.7	1.77 (1.15,2.71)
85th percentiles	24.2	1.75 (1.15,2.66)
82.5th percentiles	23.7	1.72 (1.14,2.61)
80th percentiles	23.1	1.70 (1.13,2.56)
77.5th percentiles	22.6	1.68 (1.12,2.52)
75th percentiles	22.1	1.66 (1.12,2.48)
72.5th percentiles	21.3	1.64 (1.11,2.42)
70th percentiles	20.7	1.62 (1.11,2.38)

Supplemental Table S10. Interactive and stratified analyses of temperature and $PM_{2.5}$ on pulmonary tuberculosis.[a]

Temperature	$PM_{2.5}$		Effect of $PM_{2.5}$ within strata of temperature	RERI (95% CI)	IRR (95% CI)
	Low	High			
Cold	1.00 (Reference)	1.06 (0.91, 1.24)	1.06 (0.91, 1.24)		
Heat	1.17 (1.03, 1.33)*	1.06 (0.93, 1.21)	0.91 (0.83, 0.99)	-0.17 (-0.79, 0.46)	1.06 (0.93, 1.21)
Effect for temperature within strata of $PM_{2.5}$	1.17 (1.03, 1.33)*	1.00 (0.89, 1.13)			

$PM_{2.5}$, fine particulate matter, particulate matter with aerodynamic diameters ≤ 2.5 μm; RERI, relative excess risk due to interaction; IRR, interaction relative risk.

[a] Temperature and $PM_{2.5}$ were dichotomized to create an interaction term. Temperature was categorized as cold (< minimum mortality temperature [MMT] of 1.1°C) or heat (≥ MMT). $PM_{2.5}$ was classified as low (< median concentration [29.4 μg/m³]) or high (≥ median). Asterisks denote statistically significant effects.

Supplemental Figures

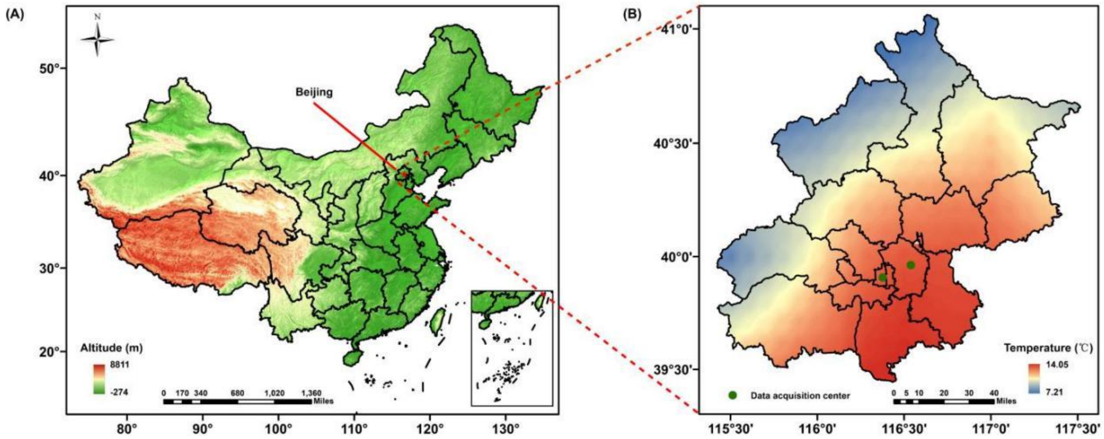

Supplemental Figure S1. Geographical location and climate characteristics of Beijing.

[a] Figure shows (A) Geographical location of Beijing in China and (B) the ambient mean temperature distribution during the study period. Green and red gradients indicate the altitudes, and Beijing is marked by the asterisk in Figure 1A. Red and blue gradients indicate the average temperature in Beijing from January 1, 2019, to December 31, 2023, in Figure 1B. The green circle indicates the data sub-center of this study.

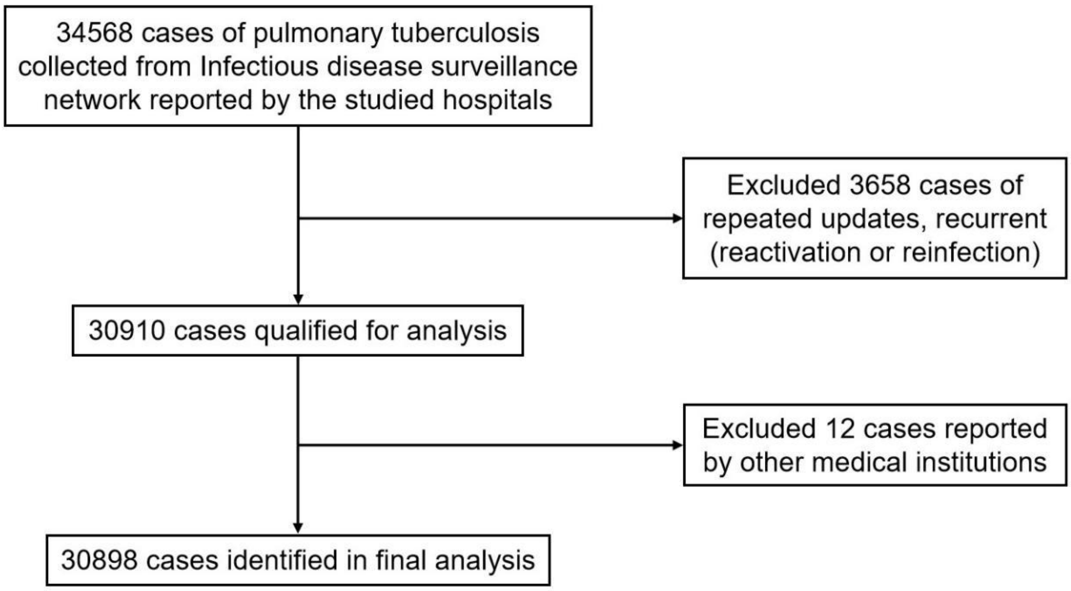

Supplemental Figure S2. Flow diagram for the inclusion and exclusion of pulmonary tuberculosis patients.

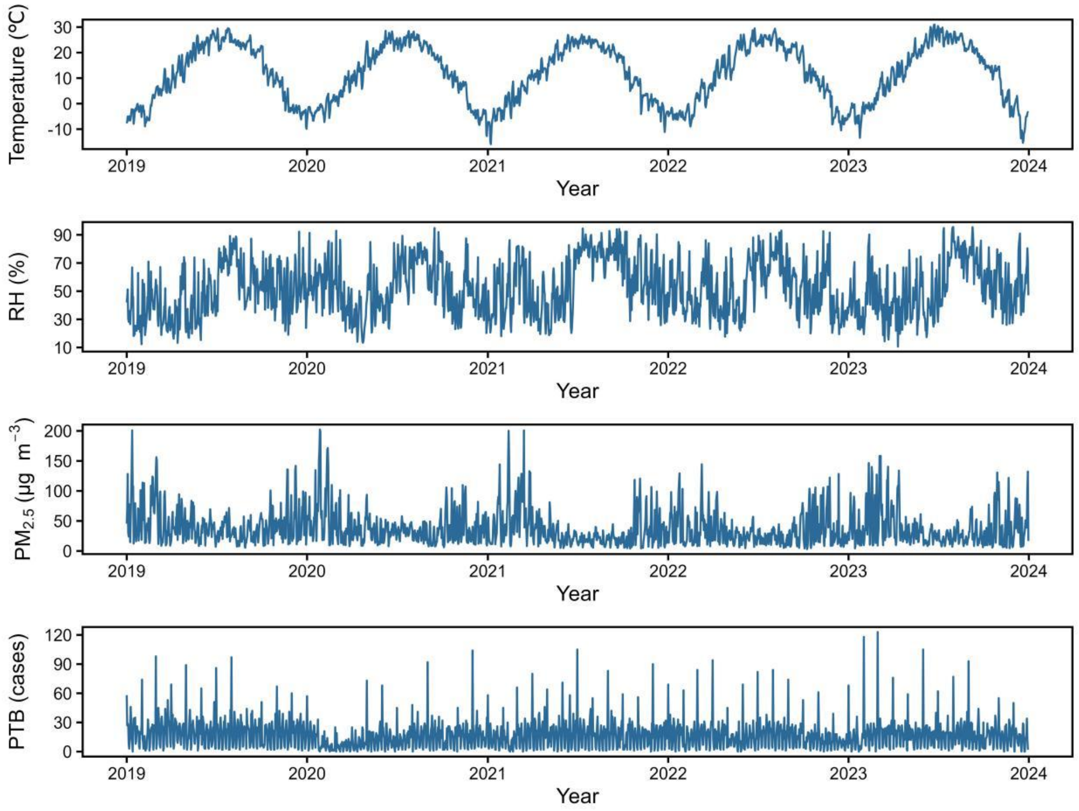

Supplemental Figure S3. Time-series plots of daily pulmonary tuberculosis events, meteorological factors, and $PM_{2.5}$ levels in Beijing, China, 2019−2023.

RH, relative humidity; $PM_{2.5}$, fine particulate matter, particulate matter with aerodynamic diameters ≤ 2.5 μm; PTB, pulmonary tuberculosis.

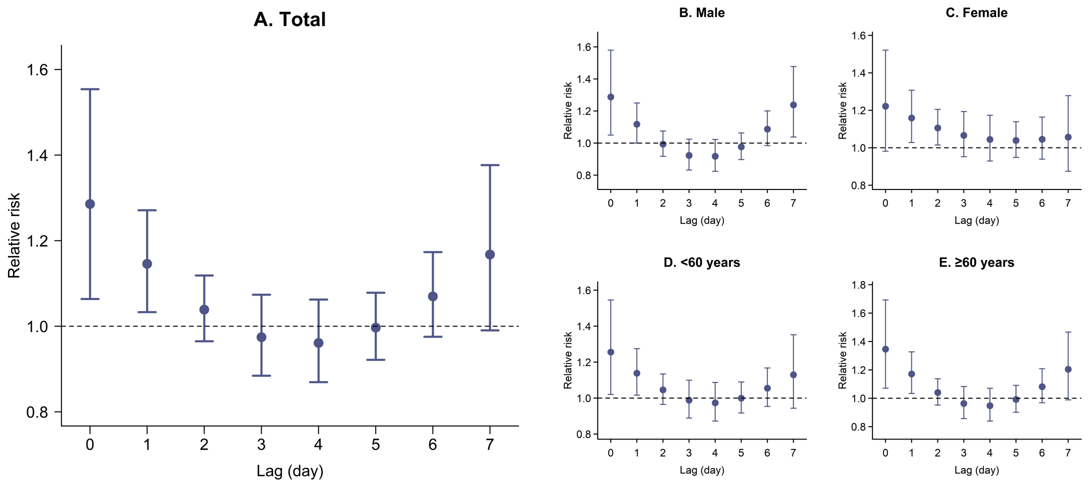

Supplemental Figure S4. Single-lag relative risks and 95% confidence intervals of pulmonary tuberculosis at sub-extremely high temperature.[a]

[a] Single lag-response curves are presented for: (A) total pulmonary tuberculosis patients; (B) males; (C) females; (D) patients aged <60 years; and (E) patients aged ≥60 years. The mean estimates of temperature-related risk attributable to sub-extremely high temperature (25.2℃, 97.5th percentile) are shown by purple lines, while shaded areas indicate the corresponding 95% confidence intervals.

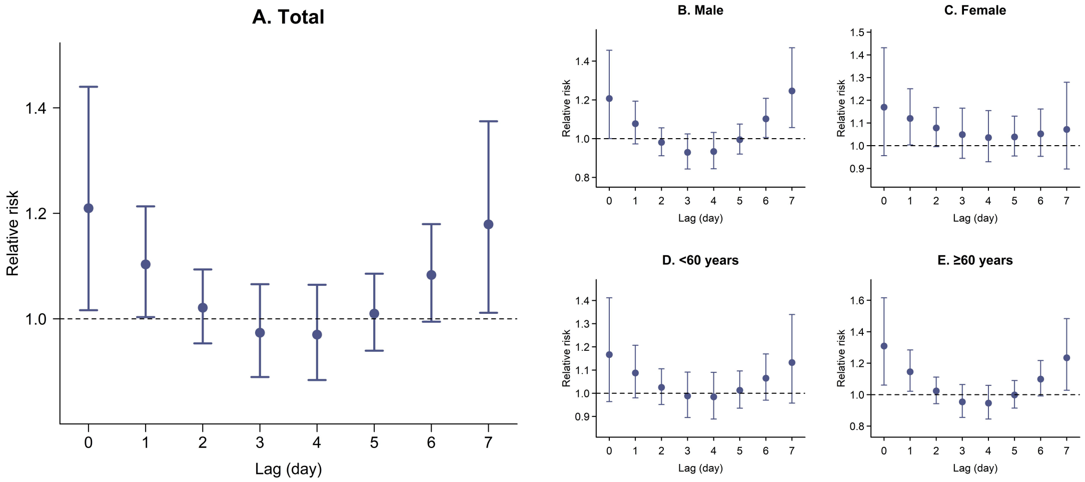

Supplemental Figure S5. Single-lag relative risks and 95% confidence intervals of pulmonary tuberculosis at moderately high temperature.[a]

[a] Single lag-response curves are presented for: (A) total pulmonary tuberculosis patients; (B) males; (C) females; (D) patients aged <60 years; and (E) patients aged ≥60 years. The mean estimates of temperature-related risk attributable to moderately high temperature (22.0℃, 97.5th percentile) are shown by purple lines, while shaded areas indicate the corresponding 95% confidence intervals.

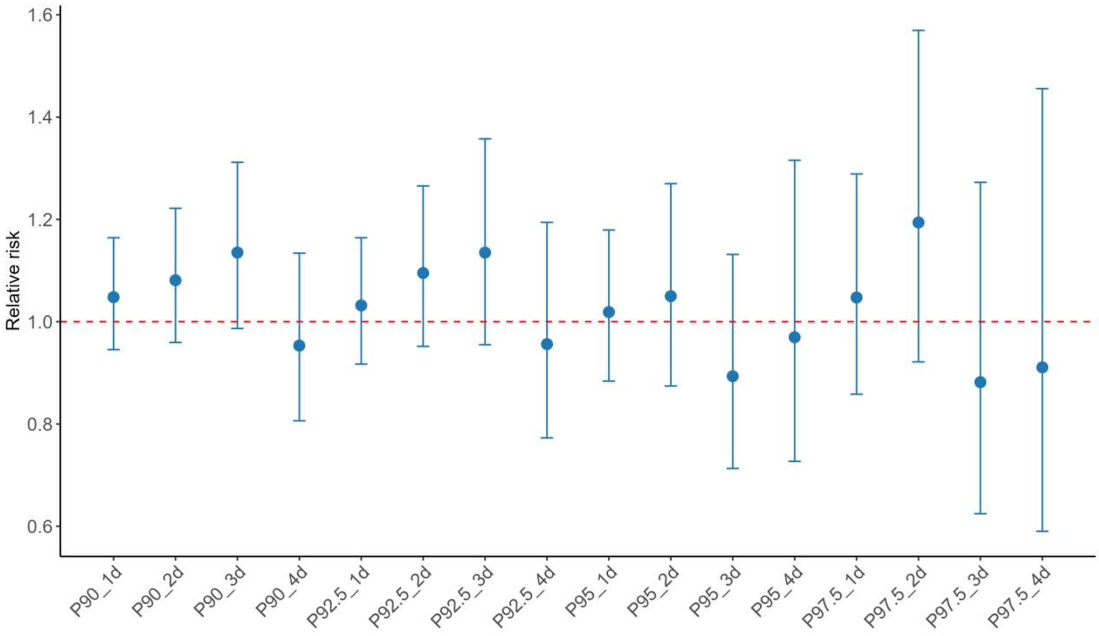

Supplemental Figure S6. Association of exposure to heat waves with pulmonary tuberculosis.[a]

[a] Heat waves are defined as periods when the daily mean temperature exceeded the 90th, 92.5th, 95th, and 97.5th percentiles for 1, 2, 3, or 4 consecutive days. Taking P95_3d as an example, it represents daily apparent temperature equal to or higher than the 95th percentile temperature for at least 3 consecutive days. The solid circle represents the relative risk, and the straight lines on both sides represent the 95 % confidence interval.

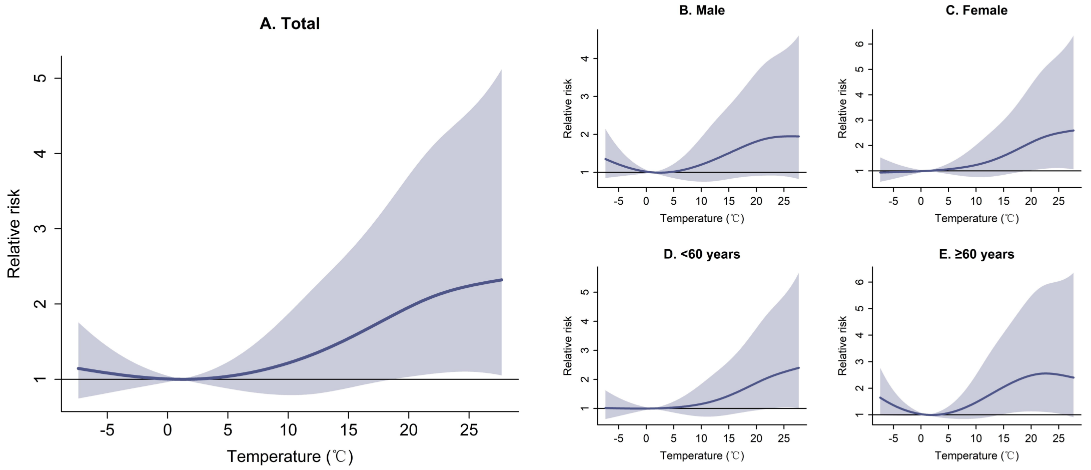

Supplemental Figure S7. Cumulative exposure-response curves between daily mean temperature and pulmonary tuberculosis risk over 0 to 7 lag days in 2019.[a]

[a] Cumulative exposure-response curves are presented for: (A) total pulmonary tuberculosis patients; (B) males; (C) females; (D) patients aged <60 years; and (E) patients aged ≥60 years. The mean estimates of temperature-related risk are shown by purple lines, while shaded areas indicate the corresponding 95% confidence intervals. The temperature range shown on the x-axis (–7.6°C to 27.7°C) reflects the truncated range (2.5th to 97.5th percentiles) used in the analytical models.

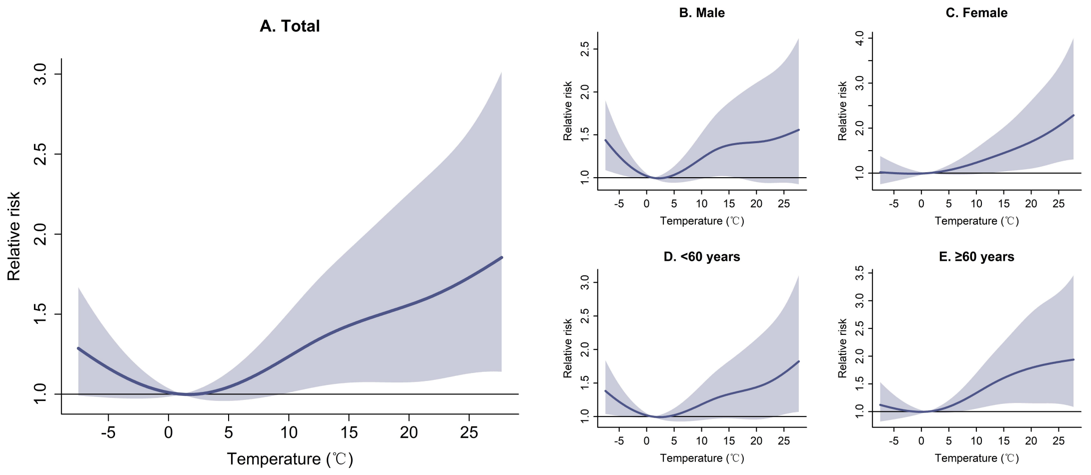

Supplemental Figure S8. Cumulative exposure-response curves between daily mean temperature and pulmonary tuberculosis risk over 0 to 7 lag days after adjusting the impact of the COVID-19.[a]

a Cumulative exposure-response curves are presented for: (A) total pulmonary tuberculosis patients; (B) males; (C) females; (D) patients aged <60 years; and (E) patients aged ≥60 years. The mean estimates of temperature-related risk are shown by purple lines, while shaded areas indicate the corresponding 95% confidence intervals. The temperature range shown on the x-axis (–7.6°C to 27.7°C) reflects the truncated range (2.5th to 97.5th percentiles) used in the analytical models. The impact of the COVID-19 was excluded by adjusting the binary variables (2019: 1; 2020-2023: 0).

References

[1]. Alduchov OA, Eskridge RE. Improved magnus form approximation of saturation vapor pressure. J Appl Meteorol, 1996, 35(4):601–9.

[2]. Chen R, Peng RD, Meng X, Zhou Z, Chen B, Kan H. Seasonal variation in the acute effect of particulate air pollution on mortality in the China Air Pollution and Health Effects Study (CAPES). Sci Total Environ, 2013, 450-451:259-65.
